# Occult hepatitis C virus infection in hemodialysis patients who achieved a sustained virological response to directly acting antiviral drugs: is it a concern?

**DOI:** 10.1007/s11255-023-03621-1

**Published:** 2023-05-20

**Authors:** Hend Naguib, Shady Fouad Abouelnaga, Mohamed Mamdouh Elsayed

**Affiliations:** 1 Hepatology and Internal Medicine Department, Faculty of Medicine, Alexandria University, Alexandria, Egypt; 2 Clinical and Chemical Pathology Department, Alexandria University Hospitals, Alexandria, Egypt; 3 Nephrology and Internal Medicine Department, Faculty of Medicine, Alexandria University, Alexandria, Egypt

**Keywords:** Direct-acting antiviral drugs, Hemodialysis, Occult hepatitis C infection (OCI), End-stage renal disease (ESRD), Sustained virological response (SVR)

## Abstract

**Purpose:**

Hepatitis C virus infection is a major health problem in hemodialysis patients. Occult HCV infection is defined as the presence of HCV-RNA in hepatocytes or peripheral blood mononuclear cells without the detection of HCV-RNA in the serum. We aimed to evaluate the prevalence and predictors of occult HCV infection among hemodialysis patients after treatment with direct-acting antiviral agents.

**Methods:**

This research is a cross-sectional study that included 60 HCV patients maintained on regular HD patients who achieved 24 weeks of sustained virological response after treatment with direct-acting antiviral agents. Real-time PCR was performed to detect HCV-RNA in peripheral blood mononuclear cells.

**Results:**

HCV-RNA was detected in peripheral blood mononuclear cells of three patients (5%). Occult HCV infection cases were treated by Interferon/ribavirin before direct-acting antiviral agents and two of them had raised pre-treatment alanine aminotransferase levels. Logistic regression analyses revealed that high pre-treatment viral load and raised pre-treatment alanine aminotransferase were associated with an increased risk of occult HCV infection with *p* value of 0.041 and 0.029, respectively.

**Conclusions:**

Occult HCV infection in hemodialysis patients who achieved sustained virological response after treatment with direct-acting antiviral agents may occur, and this may necessitate dual testing for HCV in both serum and peripheral blood mononuclear cells to ensure viral clearance.

**Clinical trials registration:**

ClinicalTrials.gov NCT04719338.

## Introduction

Hepatitis C virus (HCV) infection is an important cause of chronic liver disease and represents a major public health problem [1, 2]. In 2015, the World Health Organization (WHO) launched a strategy aiming to eliminate HCV as a worldwide health concern by 2030 [3]. Treatment of HCV infection through achieving a sustained virological response (SVR) improved dramatically with the introduction of directly acting antiviral drugs (DAADs) [4].

Occult HCV infection (OCI) is defined as the presence of HCV-RNA in hepatocytes or peripheral blood mononuclear cells (PBMCs) with no detectable HCV-RNA in the serum by the conventional PCR assays [5]. It can be diagnosed through a liver biopsy which is the gold standard method but with a higher risk of bleeding or through PCR assay in PBMCs [6].

In the hemodialysis (HD) population, HCV infection represents a major problem with higher prevalence and poorer outcomes than the general population [7, 8]. This higher prevalence is due to prolonged vascular access, contact with contaminated equipment, frequent blood transfusion, and poor adherence to infection control measures [9, 10]. OCI was found in HD patients [11, 12] and in persons who achieved SVR to DAADs but were not on dialysis [13, 14]. OCI may represent a higher risk for disease relapse, progression, and transmission of infection to others. To our knowledge, this is the first research to address the problem of OCI in HD patients who achieved SVR after treatment with DAADs.

## Materials and methods

### Participants

This research is a cross-sectional study that enrolled 60 patients maintained on regular HD from two dialysis units in Alexandria for more than 3 months who achieved SVR 24 weeks after the end of treatment with DAADs. Qurevo (ombitasvir/paritaprevir/ritonavir) with ribavirin was used as it is the approved protocol to treat HCV in ESRD patients in Egypt. They perform thrice weekly, 4 h HD sessions. Patients were either treatment-naive or experienced to previous Interferon/RBV regimens. The trial was registered on Clinicaltrials.gov (NCT04472637).

### Methods and study outcomes

All patients were subjected to full history taking and thorough clinical examination. We assessed the initial pre-treatment data of the patients including pre-treatment status (naïve, experienced), viral load by sensitive real-time HCV-PCR technique, serum alanine aminotransferase (ALT), albumin, bilirubin, prothrombin time, and Child–Pugh score.

Detection of HCV-RNA in PBMCs was done by quantitative real-time PCR. Peripheral blood samples (7 ml) were obtained in two EDTA-containing tubes. After plasma separation, the layer of PBMCs was isolated by Ficoll-Paque gradient centrifugation, washed with phosphate buffer saline (PBS), and resuspended. Quantification and detection of HCV virus in the specimens were done using the COBAS AmpliPrep/COBAS TaqMan HCV test (P/N: 03568555 190) with the automated COBAS TaqMan 48 analyzer (ROCHE Diagnostics). The process is based on three major steps: (1) specimen preparation to isolate HCV-RNA; (2) reverse transcription of the target RNA to cDNA, and (3) PCR amplification of target cDNA and detection.

### Statistical analysis

All statistical analyses were conducted by IBM SPSS software package version 20.0. (Armonk, NY: IBM Corp). Categorical data were represented as numbers and percentages. *χ*^2^ test was applied to investigate the association between the categorical variables. Alternatively, Monte Carlo or Fisher’s exact correction test was applied when the expected cell counts were less than 5. Distributed data were expressed as a range (minimum and maximum), mean, standard deviation, and median. Student’s *t *test was used to compare two groups for normally distributed quantitative variables. The significance of the obtained results was judged at the 5% level. Univariate logistic regression analyses were performed to analyze factors associated with positive HCV-PCR in PMNCs.

## Results

In total, 60 HD patients were enrolled in the study with ages ranging from 40 to 61 years; 32 of the patients were males. Forty-five patients were treatment-naive and 15 of them were treatment-experienced. The findings showed that out of the 60 patients, three of them (5%) were proved to have occult HCV infection (positive HCV-PCR PBMCs). The three patients had different causes for ESRD (hypertension, diabetes mellitus, and analgesic nephropathy). Patients with negative HCV-PCR PBMCs and patients with positive HCV-PCR PBMCs were matched according to age, gender, and vintage of HD. The pre-treatment serum ALT level was significantly higher among patients with positive HCV-PCR PBMCs compared to patients with negative HCV-PCR PBMCs (*p* = 0.044). All patients with positive HCV-PCR PBMCs were treatment-experienced. The average pre-treatment viral load was significantly higher among patients with positive HCV-PCR PBMCs compared to patients with negative HCV-PCR PBMCs (6.07 ± 0.95 versus 4.23 ± 1.14 log 10 IU/ml) (*p* = 0.008), as shown in Table 1.

**Table 1:** Clinical characteristics and laboratory parameters of patients

	Total (*n* = 60)	HCV-PCR in PBMCs		*P* value
		Negative HCV-PCR in PBMCs (*n* = 57)	Positive HCV-PCR in PBMCs (*n* = 3)	
Sex				
Male	32 (53.3%)	31 (54.4%)	1 (33.3%)	0.594
Female	28 (46.7%)	26 (45.6%)	2 (66.7%)	
Age (years)				
Mean ± SD	51.60 ± 5.06	51.6 ± 5.2	51.33 ± 3.21	0.926
Median (Min.–Max.)	50 (40–61)	50 (40–61)	50 (49–55)	
Vintage of HD (years)				
Mean ± SD	8.67 ± 7.62	8.62 ± 7.48	8.18 ± 5.65	0.458
Median (Min.–Max.)	5.3 (0.5–15)	5.3 (0.5–15)	6 (1.25–11)	
Pre-treatment status				
Naïve	45 (75%)	45 (78.9%)	0 (0%)	0.013
Experienced	15 (25%)	12 (21.1%)	3 (100%)	
Pre-treatment viral load (10^6^ IU/ml)				
Mean ± SD	4.32 ± 1.20	4.23 ± 1.14	6.07 ± 0.95	0.008*
Median (Min.–Max.)	4.4 (2.20–7.0)	4.30 (2.20–6.2)	6.10 (5.1–7.0)	
Serum ALT (u/l)				
Pre-treatment				
Normal	52 (86.7%)	51 (89.5%)	1 (33.3%)	0.044*
Raised	8 (13.3%)	6 (10.5%)	2 (66.7%)	
Post-treatment				
Normal	57 (95%)	55 (96.5%)	2 (66.7%)	0.145
Raised	3 (5%)	2 (3.5%)	1 (33.3%)	
Serum bilirubin (mg/dl)				
Pre-treatment				
Mean ± SD	1.3 ± 0.49	1.3 ± 0.47	0.85 ± 0.67	0.106
Median (Min.–Max.)	1.5 (0.20–2.1)	1.5 (0.60–2.1)	0.80 (0.20–1.5)	
Post-treatment				
Mean ± SD	0.88 ± 0.26	0.91 ± 0.21	0.32 ± 0.48	0.164
Median (Min.–Max.)	0.93 (0.01–2)	0.93 (0.50–2)	0.07 (0.01–0.87)	
Serum albumin (g/dl)				
Pre-treatment				
Mean ± SD	3.8 ± 0.45	3.7 ± 0.44	4 ± 0.68	0.405
Median (Min.–Max.)	3.8 (2.8–4.5)	3.8 (2.8–4.5)	4.2 (3.2–4.5)	
Post-treatment				
Mean ± SD	3.7 ± 0.29	3.7 ± 0.28	3.8 ± 0.35	0.460
Median (Min.–Max.)	3.6 (3.2–4.6)	3.6 (3.2–4.6)	3.8 (3.5–4.2)	
Prothrombin time				
Pre-treatment				
Normal	26 (43.3%)	25 (43.9%)	1 (33.3%)	1.000
Prolonged	34 (56.7%)	32 (56.1%)	2 (66.7%)	
Post-treatment				
Normal	29 (48.3%)	29 (50.9%)	0 (0%)	0.238
Prolonged	31 (51.7%)	28 (49.1%)	3 (100%)	
KT/V Daugirdas				
Pre-treatment				
Mean ± SD	1.34 ± 0.67	1.34 ± 0.53	1.36 ± 0.32	0.538
Median (Min.–Max.)	1.34 (0.9–1.75)	1.34 (0.9–1.75)	1.36 (1.26–1.37)	
Post-treatment				
Mean ± SD	1.41 ± 0.45	1.41 ± 0.69	1.38 ± 0.29	0.349
Median (Min.–Max.)	1.40 (1–1.82)	1.40 (1–1.82)	1.37 (1.32–1.5)	
Child–Pugh class pre-treatment				
Non-cirrhotic	22 (36.7%)	22 (38.6%)	0 (0%)	0.191
A	18 (30%)	16 (28.1%)	2 (66.7%)	
B	20 (33.3%)	19 (33.3%)	1 (33.3%)	

Data were expressed as mean ± standard deviation (SD), Median (Min.–Max.), or absolute numbers as appropriate. *p*: *p* value for comparing between the studied groups

*ALT* alanine aminotransferase, *HCV* hepatitis C virus, *Kt/V* measuring dialysis adequacy, *PBMCs* peripheral blood mononuclear cells, *PCR* polymerase chain reaction

*Statistically significant at *p* ≤ 0.05

There were no significant differences between patients of both groups regarding pre-treatment laboratory findings including prothrombin time, serum albumin, bilirubin levels, and KT/V. Post-treatment prothrombin time, serum ALT, bilirubin, albumin levels, and KT/V did not show any statistical differences between the two groups. Also, there was no difference regarding the pre-treatment Child–Pugh score (*p* > 0.05) as shown in Table 1.

Logistic regression analysis for prediction of OCI revealed the following risk factors (OR): raised pre-treatment viral load (OR = 10.003, *p* = 0.041) and raised pre-treatment ALT level (OR = 17.0, *p* = 0.029), as shown in Table 2.

**Table 2:** Univariate analysis of factors associated with occult HCV infection

Positive HCV-PCR in PMNCs	Univariate	
	*p*	OR (95%CI)
Pre-treatment viral load	0.041*	10.003* (1.093–91.524)
Serum alanine aminotransferase (ALT)		
Pre-treatment	0.029*	17.0* (1.334–216.66)
Post-treatment	0.065	13.750 (0.849–222.61)

*OR* odd’s ratio, *CI* confidence interval

^#^All variables with *p* < 0.05 were included; *statistically significant at *p* ≤ 0.05

## Discussion

This study is the first research to evaluate the prevalence and predictors of OCI among HD patients who achieved SVR after treatment with DAADs. OCI patients may carry a potential risk for disease relapse, disease progression, and higher rate of liver fibrosis [13]. Moreover, it can be potentially infectious and could be the offender of HCV spread [15]. Afterward, the term OCI represents the greatest challenge against the argument of SVR [13]. Thus, careful attention must be paid to the diagnosis of OCI. This is especially important among HD patients, because HCV reactivation may increase morbidity and mortality risks [8]. Our main finding was that 5% of the included patients had OCI. These results are in accordance with the results obtained by a previous study conducted by Yousif et al., who tested OCI among different regimens of oral treatment and found that the prevalence of OCI was 12%, but they included only patients with normal kidney functions [14]. These results are similar to that conducted by Abd Alla and El Awady who reported a considerably higher prevalence of OCI (18%) using PBMCs’ PCR in sustained responders to DAAs, but they did not provide data about kidney functions’ status [16].

Another study detected about 4% of OCI in patients with SVR12 [13]. Ibarra et al. documented the impaired cellular uptake of RBV into PBMCs with time. This may provide an explanation for the question of why PBMCs could become a reservoir of HCV [17].We suggest that those patients should be followed up carefully with more frequent investigations to discover if OCI in HD patients can cause reactivation and flare up of the virus or can be still infectious. These concerns can be answered in future studies with longer follow-up periods.

In our study, there were statistically significant differences between patients with positive HCV-PCR PBMCs and patients with negative HCV-PCR PBMCs regarding pre-treatment status (*p* = value 0.013). These results were in concordance with that reported by Mekky et al. who reported that all OCI patients were treatment-experienced [13]. Conflicting results were found in the literature. Abd Alla et al. reported a high prevalence of OCI among treatment-experienced and naive cases [16].

Regarding serum alanine aminotransferases (ALT) prior to treatment, our study revealed that there was statistically significant difference between patients with positive HCV-PCR PBMCs and patients with negative HCV-PCR. These results are in line with the results obtained by the previous study [13]. Also, we found that post-treatment serum alanine aminotransferases ALT did not show any statistical difference between the two groups. On the other hand, Elmasry et al. studied the occurrence of OCI in 134 patients who received DAAs and achieved SVR12 after liver transplantation. They found altered aminotransferase levels post-DAAs’ therapy in 11% of these OCI patients. Their results may be explained by the consequence of concomitant usage of immunosuppressive regimens [18].

In the current study, logistic regression analysis revealed that raised pre-treatment viral load and raised pre-treatment ALT level were associated with the increased risk of OCI. Similar findings were reported by Mekky et al. [13] who found that high pre-treatment viral load and raised ALT prior to treatment were the most significant predictor for the possibility of OCI presence with Odds Ratio of 7.03 and 5.13, respectively.

The strength of our study includes being the first to address the occurrence of OCI in ESRD patients on maintenance hemodialysis who achieved SVR after DAADs in a considerable number of patients (*n* = 60). The main limitation of this study might be the lack of genotype testing. Moreover, most of the studies documented genotype 4 as the main genotype in Egypt. Also, we did not test for OCI in liver biopsy. Despite that PBMCs test underestimates the true prevalence of OCI and only 70% of OCI cases can be detected, liver biopsy is not always available, and it carries a lot of risks, such as bleeding or inadvertent another organ puncture. Another point, the number of patients with OCI (*n* = 3) may be too small for extracting safe conclusions about the predictive factors (ALT, viral load), but this is expected given this new condition and allows for future, larger research to be conducted to investigate this problem.

## Conclusion

In conclusion, OCI can be detected within a considerable number of patients with apparent clearance of HCV-RNA in serum. These findings might suggest the utilization of dual testing for HCV-RNA in both serum and PBMCs as a routine part of post-treatment workup especially in localities with a high HCV endemicity for at least those with abnormal pre-treatment aminotransferases. Larger studies are needed to confirm these findings.

## Data Availability

All data analyzed during this study are included in this manuscript.
